# Salvianolic Acid B Inhibits Ferroptosis and Apoptosis during Myocardial Ischemia/Reperfusion Injury via Decreasing the Ubiquitin-Proteasome Degradation of GPX4 and the ROS-JNK/MAPK Pathways

**DOI:** 10.3390/molecules28104117

**Published:** 2023-05-16

**Authors:** Xiaojin Xu, Chenhan Mao, Chengbo Zhang, Meng Zhang, Jianbin Gong, Xindong Wang

**Affiliations:** 1 The Third Clinical Medical College, Nanjing University of Chinese Medicine, Nanjing 210028, China; 2 Jinling Clinical Medical College, Nanjing University of Chinese Medicine, Nanjing 210023, China; 3 Affiliated Hospital of Integrated Traditional Chinese and Western Medicine, Nanjing University of Chinese Medicine, Nanjing 210028, China; 4 Jiangsu Province Academy of Traditional Chinese Medicine, Nanjing 210028, China; 5 Shuguang Hospital Affiliated to Shanghai University of Traditional Chinese Medicine, Shanghai 201203, China

**Keywords:** salvianolic acid B, myocardial ischemia/reperfusion injury (MIRI), ferroptosis, apoptosis, reactive oxygen species (ROS), GPX4

## Abstract

Myocardial ischemia/reperfusion injury (MIRI) is related to ferroptosis and apoptosis elicited by reactive oxygen species (ROS). In this research, we investigated the protective effect of salvianolic acid B (SAB) as a natural antioxidant on ferroptosis and apoptosis in the MIRI process, and discussed the protective mechanism inhibiting ubiquitin-proteasome degradation of glutathione peroxidase 4 (GPX4) and the c-Jun N-terminal kinases (JNK) apoptosis signal pathway. We observed that ferroptosis and apoptosis occurred in the MIRI rat model in vivo and the H9c2 cardiomyocyte hypoxia/reoxygenation (H/R) damage model in vitro. SAB can alleviate tissue damage related to ROS, ferroptosis and apoptosis. Ubiquitin-proteasome degradation of GPX4 occurred in H/R models, and SAB reduced the ubiquitin-proteasome degradation of GPX4. SAB downregulates JNK phosphorylation and the expression of BCL2-Associated X (Bax)/B-cell lymphoma-2 (Bcl-2) and Caspase-3 to inhibit apoptosis. The role of GPX4 in the cardioprotection of SAB was further verified by the elimination effect of the GPX4 inhibitor RAS-selective lethal 3 (RSL3). This research shows that SAB may be used as a myocardial protective agent against oxidative stress, ferroptosis and apoptosis, and has potential clinical application prospects.

## 1. Introduction

Although significant progress has been made in reperfusion therapy such as thrombolysis or percutaneous interventions (PCI), the 1-year mortality and heart failure readmission rate of patients experiencing acute ST-segment elevation myocardial infarction (STEMI) after PCI are still as high as 7~12% and 22% [1]. The process of reperfusion of the ischemic myocardium in the infarcted area was done by opening the occluded vessel after STEMI was accompanied by severe MIRI, which leads to increased myocardial dysfunction and further myocardial cell death. MIRI is an independent predictor of heart failure and poor long-term prognosis [2]. Various cardioprotective strategies have attempted to alleviate reperfusion injury, reduce hospitalization rates for heart failure, and improve prognosis, but the clinical effects are still controversial [3,4].

Induction of cardiomyocyte death during MIRI is carried out by a variety of regulated pathways such as caspase-mediated apoptosis [5], ROS-induced oxeiptosis [6], inflammasome-activated pyroptosis [7], and iron/lipid dependent ferroptosis [8] pathways. Ferroptosis is a type of iron-dependent cell death caused by the destructive amassing of lipid peroxide and ROS [9,10]. According to previous reports, ferroptosis contributes significantly to the process of MIRI [11]. GPX4 is a critical negative regulator of ferroptosis that is capable of reducing hydroperoxides [12]. The ubiquitin-proteasome system (UPS) and autophagy are the two intracellular protein degradation pathways in mammalian cells. Dysfunctional degradation systems can induce a variety of pathological processes. Depending on the substrate they degrade, the cell degradation mechanisms have a dual role in regulating ferroptosis. UPS can degrade key iron death inhibitors, such as the solute carrier family 7 member 11 (SLC7A11) and GPX4 [13]. Inactivation of GPX4 directly induces ferroptosis [14]. It is unclear whether UPS is involved in the degradation of GPX4 during MIRI, so it further induces ferroptosis. At the same time, it is uncertain whether the degradation of GPX4 further induces apoptosis while increasing ferroptosis and ROS accumulation.

The mitogen-activated protein kinases (MAPKs) signaling pathway is related to cell injury activated by ROS after MIRI [15]. The major MAPKs are extracellular signal-regulated kinases (ERKs), JNKs, and p38, which govern apoptosis, ferroptosis, and inflammation in response to stimulation [16]. ERK, JNK, and p38 protein kinases perform different physiological functions in cells. ERK can promote cell survival by inhibiting apoptosis, while JNK and p38 pathways promote cell apoptosis. While the degradation of GPX4 induces ferroptosis during MIRI, whether the accumulation of ROS activates MAPKs to induce apoptosis and aggravate myocardial injury remains to be further clarified.

**Figure 1:**
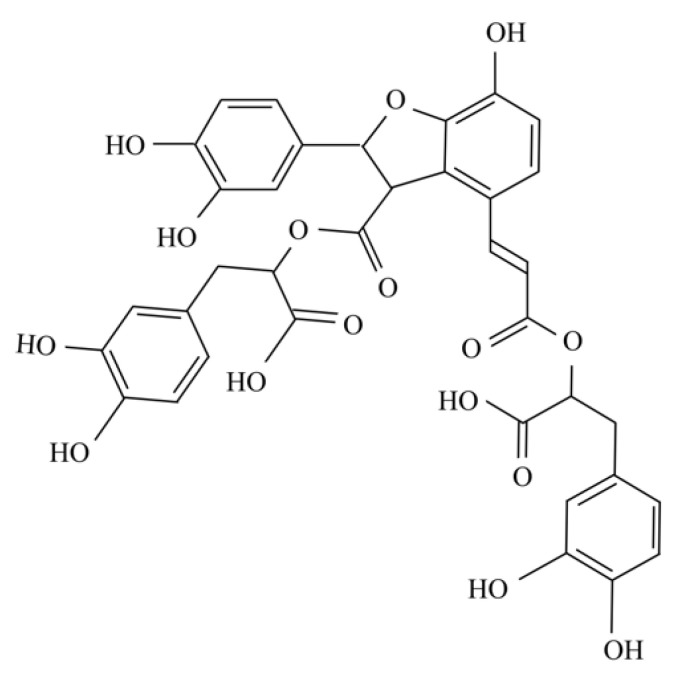
Chemical structure of Salvianolic acid B (SAB).

*Salvia miltiorrhiza Bunge* (Danshen), a well-known Chinese herbal medicine, has been extensively used in China and different Asian countries [17,18]. Danshen has the ability to activate blood and eliminate stasis according to Chinese medicine, thus having the ability to treat cardiovascular, cerebrovascular, hepatic diseases and so on [19,20,21]. Salvianolic acid B (SAB) is a water-soluble active component isolated from Danshen [22]. The molecular formula is C_36_H_30_O_16_ and its structure is shown in Figure 1. Numerous studies have shown that SAB can protect cardiomyocytes from oxidative stress-induced damage during MIRI [23,24,25]. The exact mechanisms of SAB’s protective effect on MIRI, nevertheless, are not completely grasped.

In this research, we examined the underlying mechanism of the protective effect of SAB on MIRI using a rat MIRI model and an H9c2 cardiomyocyte injury model induced by cellular H/R, demonstrating that SAB inhibits ferroptosis and ROS-activated apoptosis by regulating the ubiquitination proteasome degradation of GPX4 and the ROS-JNK/MAPK signaling pathway. This plays a protective effect on ischemia-reperfusion myocardium.

## 2. Results

### 2.1. SAB Protects Hearts from Myocardial I/R Injury in Rats

**Figure 2:**
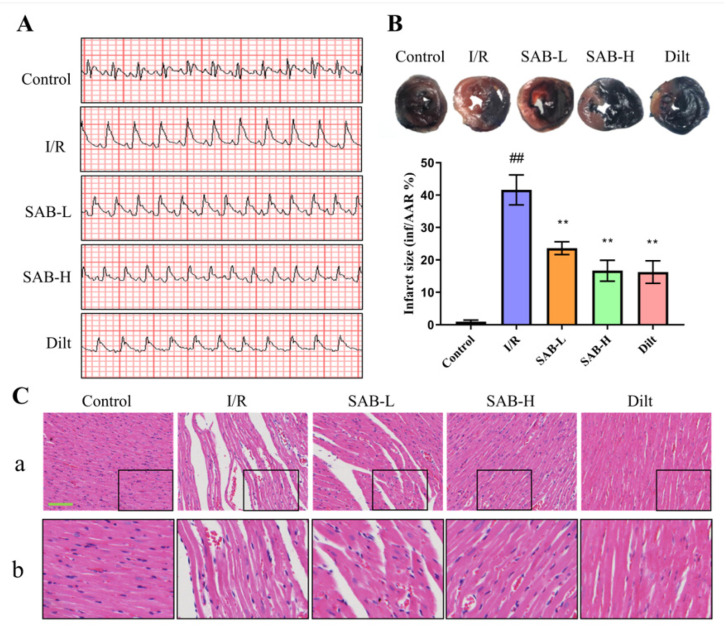
SAB prevents myocardial I/R injury in rats. (**A**) Representative images of ECG tracings in different groups at 40 min postischemic. (**B**) Representative photographs of cardiac sections by Evans Blue and TTC double staining, and quantitative analysis of the infarct size ratio (*n* = 3). (**C**) Hematoxylin and eosin staining of the myocardial tissues. a. Scale bar, 100 μm; b. enlarged images of black squares. Data are presented as means ± SD. ^##^ *p* < 0.01 vs. Control group, ** *p* < 0.01 vs. I/R group.

To assess the effect of SAB on myocardial I/R, an in vivo myocardial I/R model was established by performing the LAD ligation in SD rats. As indicated in Figure 2A, evident ST-segment elevation was observed following surgery, which confirmed the successful establishment of ischemia. Moreover, the I/R group had a larger myocardial infarct area than the control group (control: 0.9597 ± 0.4908%; I/R: 41.60 ± 4.620%; SAB-L: 23.62 ± 1.990%; SAB-H: 16.67 ± 3.243%; Dilt: 16.24 ± 3.472%; Figure 2B). The H&E staining revealed that the myocardial cells from sham-operation rats were regularly arranged without necrosis (Figure 2C). In contrast, the I/R group exhibited irregularly arranged structures and widespread necrosis and inflammatory cell infiltration. However, treatment with SAB significantly prevented I/R-induced ST-segment elevation, myocardial infarction, and pathological changes.

### 2.2. SAB Inhibits I/R-Induced Ferroptosis in the Infarcted Heart

**Figure 3:**
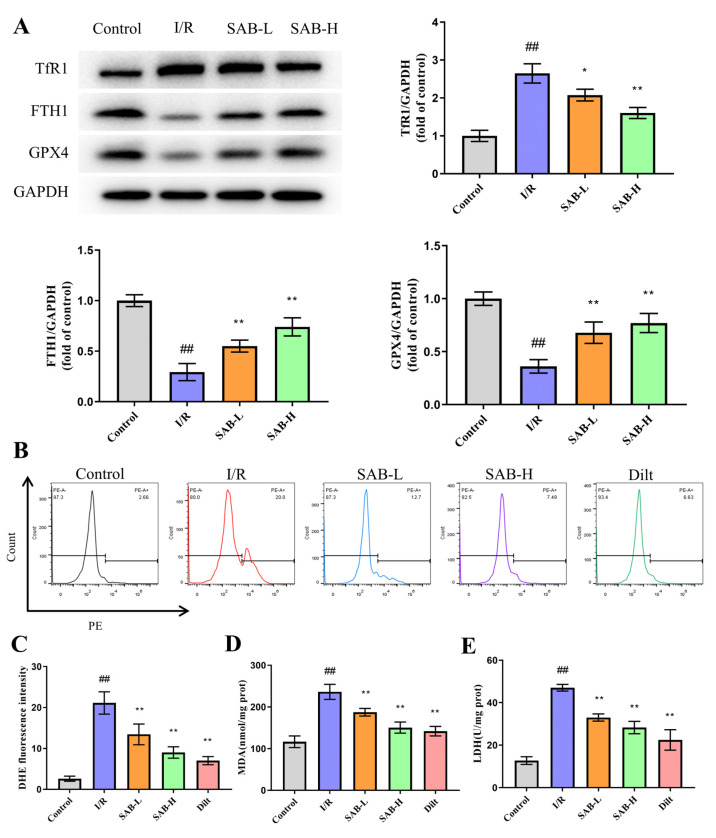
SAB reduces myocardial I/R-induced ferroptosis in the infarcted heart. (**A**) Western blot and quantitative analysis of TfR1, FTH1, and GPX4 expression in the infarcted heart. (*n* = 3) (**B**,**C**) ROS production in the infarcted heart was detected using DHE staining. (**D**,**E**) MDA and LDH levels in the infarcted heart were determined by commercial kits. (*n* = 6) Data are presented as means ± SD. ^##^ *p* < 0.01 vs. Control group, * *p* < 0.05, ** *p* < 0.01 vs. I/R group.

To identify the role of ferroptosis in the therapeutic effect of SAB in myocardial I/R damage, we assessed indexes of ferroptosis including ferroptosis-related proteins, ROS generation, and oxidative stress. Transferrin receptor 1 (TfR1) and ferritin heavy chain 1 (FTH1) are well-known regulators of intracellular iron homeostasis by controlling iron absorption and degradation, respectively [26]. The decrease of GPx4 expression is a key feature of ferroptosis. As shown in Figure 3A, in comparison with the control group, the protein expression of TfR1 in the infarcted heart was markedly increased, and the levels of FTH1 and GXP4 were dramatically decreased in the I/R group. Moreover, I/R injury resulted in a boost in ROS, MDA, and LDH levels (Figure 3B–E). Nevertheless, these effects were reversed following the SAB administration. These findings indicate that SAB ameliorates myocardial I/R-induced ferroptosis in rats.

### 2.3. SAB Decreases I/R-Induced Apoptosis in the Infarcted Heart

**Figure 4:**
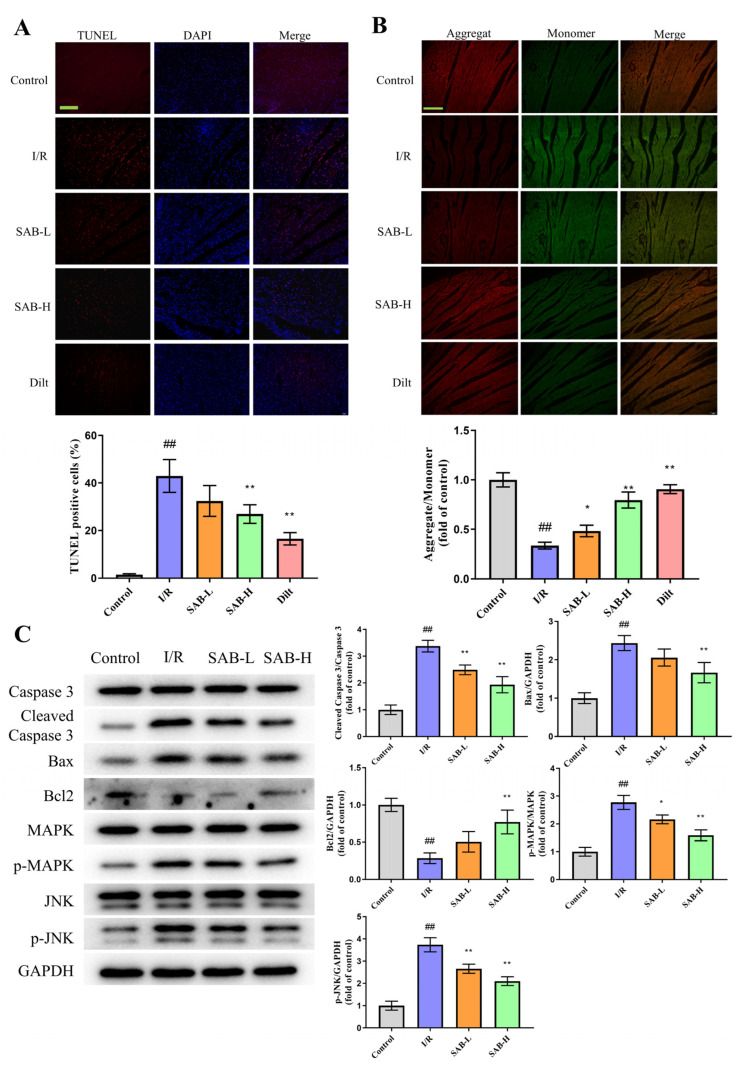
SAB suppresses myocardial I/R-induced apoptosis in the infarcted heart. (**A**) Representative images and quantitative analysis of TUNEL staining. Scale bar, 100 μm. (*n* = 3) (**B**) Representative images of JC-1 staining and quantitative analysis of the JC-1 aggregate/monomer fluorescence ratio. Scale bar, 100 μm. (*n* = 3) (**C**) Western blot and quantitative analysis of cleaved Caspase 3, Bax, Bcl2, p-MAPK, and p-JNK expression in the infarcted heart. (*n* = 3) Data are presented as means ± SD. ^##^ *p* < 0.01 vs. Control group, * *p* < 0.05, ** *p* < 0.01 vs. I/R group 3.4. SAB prevents H/R injury in H9c2 cells by regulating ferroptosis and apoptosis.

As apoptosis contributes significantly to cardiomyocyte loss and cardiac dysfunction post I/R, we investigated the in vivo effect of SAB on JNK/MAPK-mediated apoptosis [27]. As shown in Figure 4A, the number of TUNEL-positive cells (control: 1.473 ± 0.3395%; I/R: 42.93 ± 6.885%; SAB-L: 32.45 ± 6.455%; SAB-H: 26.96 ± 3.921%; Dilt: 16.57 ± 2.575%) was remarkably elevated in the I/R group relative to the controls, which was decreased by the SAB administration. The collapse of the Δψm function is known as a hallmark of apoptosis [28]. In the JC-1 staining, the loss of ΔΨm was detected in the I/R group, while SAB treatment greatly enhanced Δψm (control: 1.000 ± 0.07129; I/R: 0.3364 ± 0.03483; SAB-L: 0.4829 ± 0.05918; SAB-H: 0.7962 ± 0.08073; Dilt: 0.9055 ± 0.04494; Figure 4B). The preventive effects of SAB on apoptosis were further verified using the western blot assay. As depicted in Figure 4C, SAB downregulated cleaved Caspase 3 and Bax levels, while it upregulated Bcl2 expression by inhibiting the JNK/MAPK signaling. This confirms the involvement of JNK/MAPK-mediated apoptosis in SAB rescue of myocardial I/R damage.

### 2.4. SAB Prevents H/R Injury in H9c2 Cells through Regulating Ferroptosis and Apoptosis

**Figure 5:**
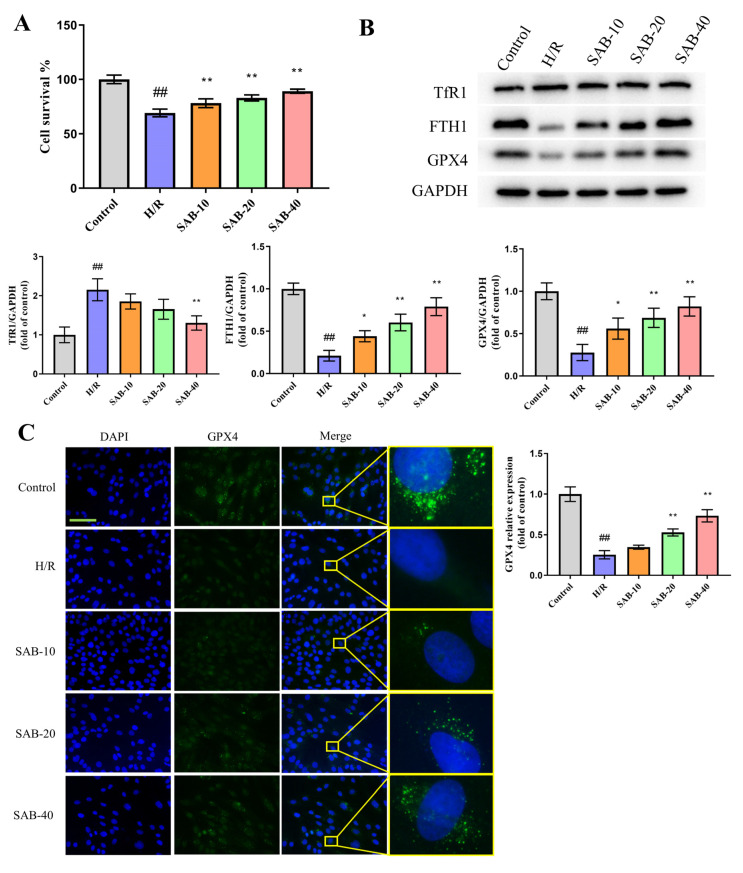

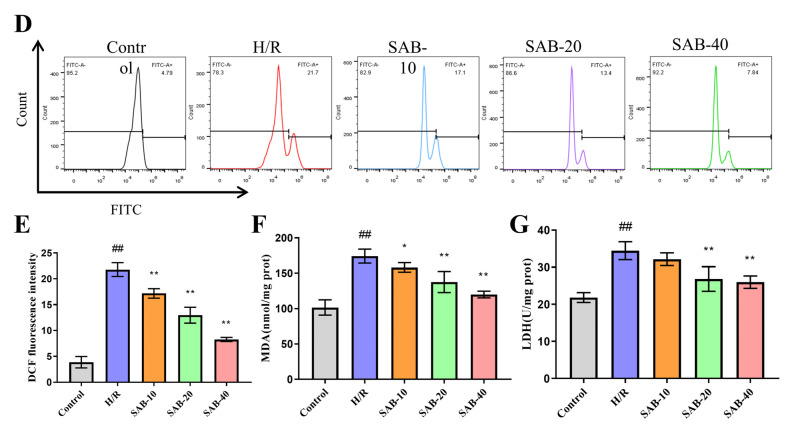
SAB improves cell survival and suppresses ferroptosis in H/R-treated H9c2 cells. (**A**) Cell survival of different groups measured by the MTT assay. (*n* = 6) (**B**) Western blot and quantitative analysis of TfR1, FTH1, and GPX4 expression in H/R-injured H9c2 cells. (*n* = 3) (**C**) Immunofluorescence staining for GPX4 in H9c2 cells. (*n* = 3) The nuclei were stained by DAPI. Scale bar, 100 μm. (**D**,**E**) ROS generation in H/R-induced H9c2 cells was detected using the DCF-DA staining. (*n* = 3) (**F**,**G**) MDA and LDH levels in H/R-exposed H9c2 cells were determined by commercial kits. (*n* = 6) Data are presented as means ± SD. ^##^ *p* < 0.01 vs. Control group, * *p* < 0.05, ** *p* < 0.01 vs. H/R group.

**Figure 6:**
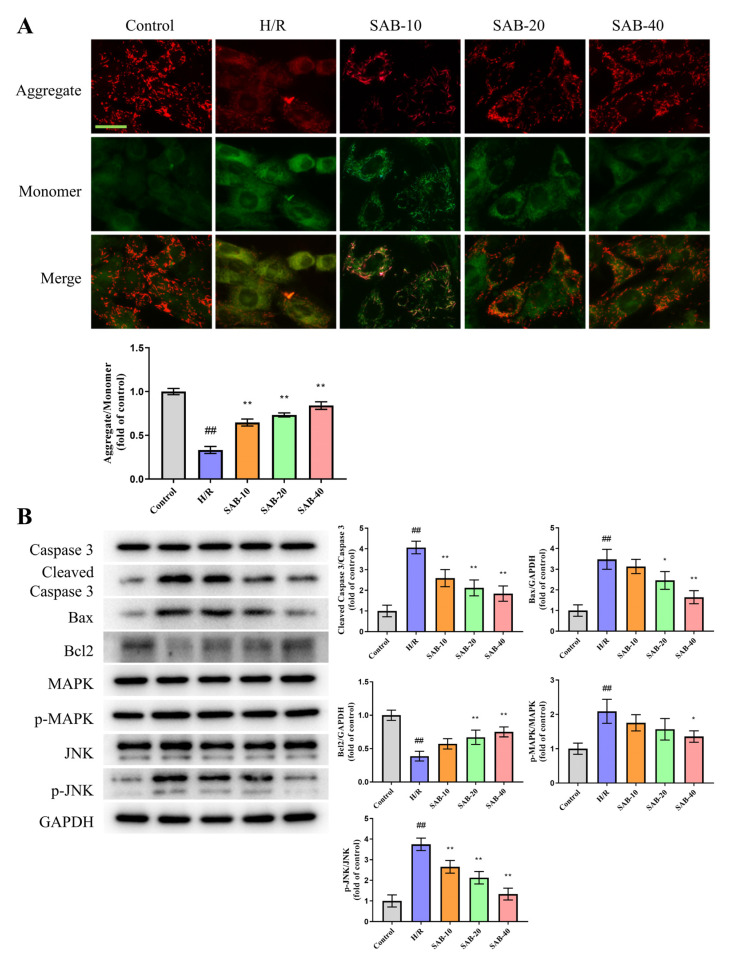
SAB prevents apoptosis in H/R-treated H9c2 cells. (**A**) Representative images of JC-1 staining and quantitative analysis of the JC-1 aggregate/monomer fluorescence ratio in H9c2 cells. (*n* = 3) Scale bar, 100 μm. (**B**) Western blots and quantitative analysis of cleaved Caspase 3, Bax, Bcl2, p-MAPK, and p-JNK expression in the infarcted heart. (*n* = 3) Data are presented as means ± SD. ^##^ *p* < 0.01 vs. Control group, * *p* < 0.05, ** *p* < 0.01 vs. H/R group.

To investigate whether SAB confers a protective effect in H/R injury in vitro, H9c2 cells were pretreated with SAB for 24 h, and then stimulated by H/R stimulation. The MTT assay was used to detect cell survival. A dose-dependent increase in cell survival (control: 100.0 ± 3.933%; H/R: 69.12 ± 3.541%; SAB-10: 78.13 ± 4.023%; SAB-20: 83.01 ± 2.824%; SAB-40: 89.19 ± 1.836%) was observed in H9c2 cells upon treatment with SAB, suggesting the protective role of SAB in H/R injury (Figure 5A). The mechanism study revealed that, consistent with the in vivo results, SAB improved H/R-caused abnormalities in ferroptosis-related protein levels (TfR1, FTH1, and GPX4) and the GPX4 fluorescent signal, while it lowered the generation of ROS, MDA and LDH in H9c2 cardiomyocytes, reflecting its effect on decreasing H/R-triggered ferroptosis (Figure 5B–G). Meanwhile, the weakened Δψm (control: 1.000 ± 0.03606; H/R: 0.3333 ± 0.04041; SAB-10: 0.6467 ± 0.04041; SAB-20: 0.7333 ± 0.02309; SAB-40: 0.8400 ± 0.04359) and apoptosis-related protein expression (cleaved Caspase 3, Bax and Bcl2) were reversed by SAB intervention through deactivating the JNK/MAPK pathway, suggesting the inhibitory role of SAB in preventing apoptosis in H/R-exposed H9c2 cells (Figure 6). We also found SAB could prevent ferroptosis and apoptosis in H/R-treated isolated adult rat cardiomyocytes (Figure S1).

### 2.5. SAB Decreases the Ubiquitin-Proteasome Degradation of GPX4 in H/R-Stimulated H9c2 Cells

**Figure 7:**
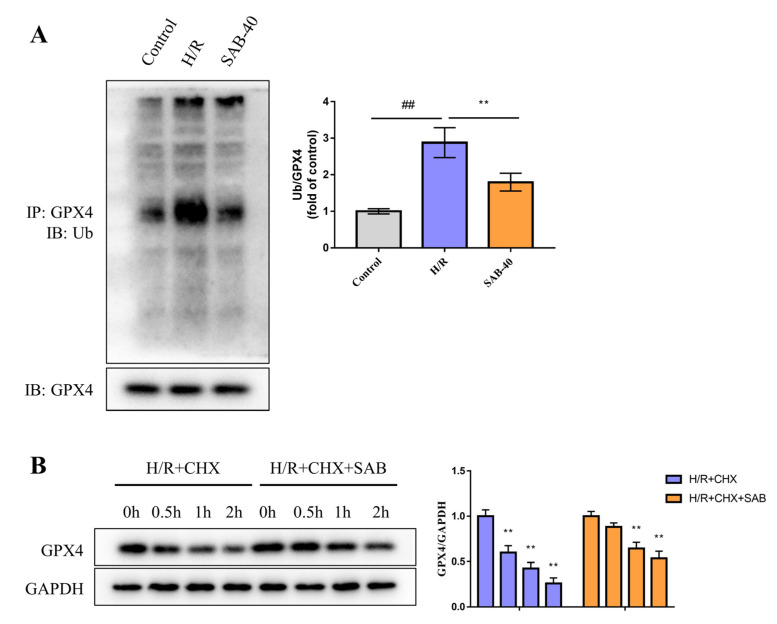
SAB decreases the ubiquitin-proteasome degradation of GPX4 in H/R-stimulated H9c2 cells. (**A**) The expression levels of ubiquitinated GPX4 were determined by co-immunoprecipitation analysis. (*n* = 3) (**B**) H9c2 cells were treated with cycloheximide for the indicated periods. GPX4 expressions were detected by the western blot assay. (*n* = 3) Data are presented as means ± SD. ^##^ *p* < 0.01, ** *p* < 0.01.

Ubiquitination is a key determinator of protein stability by tagging proteins for proteasomal degradation [29]. Herein, the role of SAB in the ubiquitin-proteasome degradation of GPX4 was investigated. As shown in Figure 7A, H/R stimulation promoted the ubiquitination of GPX4, which was significantly reversed by treatment with SAB. The cycloheximide chase assay showed that the half-life of GPX4 was much longer in the H/R + CHX + SAB group relative to the H/R + CHX cells (Figure 7B). Combined, our data indicate that SAB prevented the ubiquitin-proteasome degradation of GPX4 in H/R-treated H9c2 cells.

### 2.6. GXP4 Inhibition Abolishes the Protective Effect of SAB in H9c2 Cells Stimulated with H/R

**Figure 8:**
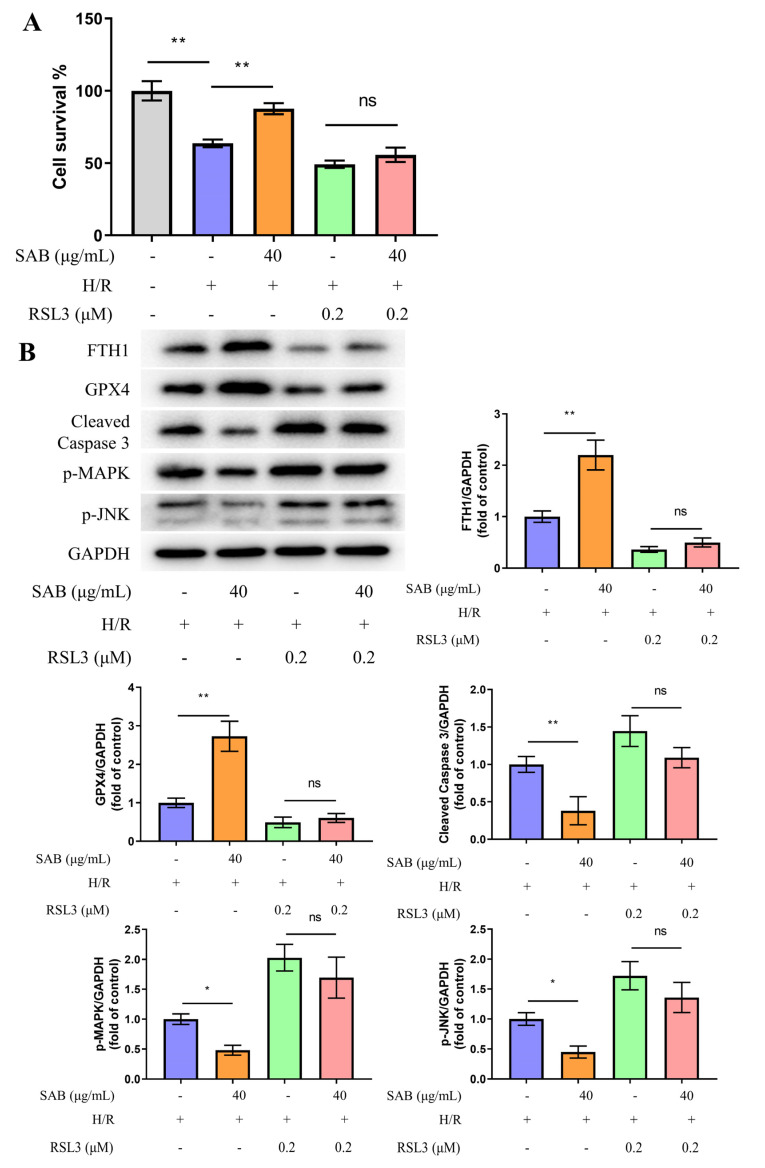
GPX4 inhibition abolished SAB-induced protective effects in H9c2 cells exposed to H/R injury. (**A**) Cell survival of different groups measured by the MTT assay. (*n* = 6) (**B**) Protein expression levels of FTH1, GPX4, cleaved Caspase 3, p-MAPK, and p-JNK were determined by western blot analysis. (*n* = 6) Data are presented as means ± SD. * *p* < 0.05, ** *p* < 0.01; ns, not significant.

Given GPX4 is crucial for the occurrence of ferroptosis and apoptosis by influencing ROS generation, the role of GPX4 in SAB-exerted cardioprotection was verified by using RSL3, a GPX4 inhibitor [16,30]. As indicated in Figure 8, SAB greatly improved cell survival and reversed the expression levels of ferroptosis- and apoptosis-associated proteins. However, RSL3 completely abrogated the aforementioned effect. These results suggested that GPX4 is a primary factor involved in SAB-exerted cardioprotective effects.

## 3. Discussion

The mechanism by which MIRI-induced cellular injury further leads to myocardial dysfunction is complex, involving reperfusion-induced cellular ROS generation, increased oxidative stress, and activation of various downstream transcription factors [31]. Therefore, inhibition of oxidative stress-related cardiomyocyte injury holds great promise for the prevention and treatment of MIRI. There is increasing evidence that SAB is a potent antioxidant, which has been demonstrated to significantly alleviate MIRI in a dose-dependent manner [23]. This study is the first to demonstrate that SAB can play a protective role for the cardiovascular system by inhibiting ferroptosis and apoptosis of cardiomyocytes during MIRI by reducing the ubiquitin-proteasome degradation of the GPX4 pathway and revealing the GPX4/ROS/JNK-mediated mechanism of crosstalk.

According to the present research, we first evaluated the effect of SAB on the I/R rat model and the H/R cell model. Diltiazem is a representative non-dihydropyridine calcium antagonist which has been widely used in the treatment of ischemic heart disease and hypertension [32]. It has an anti-myocardial ischemia-reperfusion injury effect [33,34,35], and this study was used to compare efficacies. The results showed that SAB treatment had cardioprotective effects on both in vivo model rats and in vitro model cells. In vivo, the results of TTC staining, HE staining, and the myocardial enzyme assay showed that SAB could eliminate the increase in infarct size, structural abnormalities, myocardial enzyme (LDH) elevation, and the enhanced antioxidant capacity. There was no significant difference in the infarct size ratio, the number of TUNEL-positive cells, Δψm, or the level of MDH and ROS production between the SAB-H group and the Dilt group. This means that high doses of SAB can show similar efficacy as diltiazem. Similar results were obtained in in vitro cell models. These results suggest that SAB increases myocardial viability and preserves cardiac structure after MIRI. Therefore, we further investigated and dissected the mechanisms around ROS-related ferroptosis and apoptosis to reveal the potential beneficial clinical applications of SAB.

During ferroptosis, iron accumulation and lipid peroxidation are two essential signals that activate membrane oxidative damage. TFR1 and ferritin are key regulatory points in the iron metabolism pathway. The transferrin receptor TFR1, which is located on the plasma membrane, takes up the transporter transferrin by endocytosis, so TfR1 is considered a marker protein for the occurrence of ferroptosis [36]. Enhanced expression of TFR1 increases cellular iron uptake, thereby enhancing cellular sensitivity to ferroptosis. Inhibition of TFR1 expression reduces intracellular iron content, which is beneficial to cell tolerance to ferroptosis. Ferritin is composed of FTH1, FTL, and Fe^3+^, and inhibiting the expression of FTH1 can increase the sensitivity of cells to ferroptosis. Both our in vivo and in vitro experiments indicate that I/R increased the expression of ferroptosis in cardiomyocytes, manifested as low expressions of GPX4 and FTH1, and high expression of TfR1. After the intervention of SAB, the expressions of GPX4, ROS, TfR1, and FTH1 were improved in I/R rats in vivo and in H/R cardiomyocytes in vitro, indicating that SAB can regulate the ferroptosis marker gene GPX4 and iron homeostasis regulator genes TfR1 and FTH1. These results suggest that SAB protects I/R myocardium from ferroptosis and reduces ferroptosis sensitivity by regulating GPX4, TfR1, and FTH1.

Previous studies [37] have shown that mitochondrial homeostasis is a key target of cardiac I/R injury. The mitochondrial fission and the opening of the mitochondrial permeability transition pore cause an overproduction of ROS. GPX4 inhibition was shown to lead to increased mitochondrial ROS production [38]. The present study shows that the MMP of cardiomyocytes decreased significantly after I/R injury. In contrast, SAB intervention can reduce ROS production during I/R injury and increase MMP. Accumulation of ROS can initiate apoptotic signals and aggravate myocardial injury. The intensity and balance of MAPKs activity are significant determinants of cardiac cell fate when I/R injury occurs. ERKs are activated by various growth factors which regulate cell growth and promote cell survival in I/R injury [39].We also investigated how SAB treatment affected ERK1/2 activation (Figure S2). On the contrary, p38 MAPK and JNK are activated by stress conditions and promote apoptosis by regulating the transcription of downstream factors and up-regulating the expression of apoptotic proteins or affecting mitochondrial apoptosis [40]. We further investigated the possible regulatory role of SAB in inhibiting the pro-apoptotic function of JNK under the stimulation of I/R injury. We observed that MIRI caused substantial growth in the number of TUNEL-positive cells in vitro and in vivo, and up-regulation of Bax (pro-apoptotic factor)/Bcl-2 (anti-apoptotic factor) and Caspase-3 expression increased cardiomyocyte apoptosis. SAB inhibited I/R-induced apoptosis by down-regulating the expressions of Bax/Bcl-2 and Caspase-3. Our study found that SAB inhibited the pro-apoptotic signaling pathway of JNK/MAPK activated by ROS accumulation after I/R mitochondrial injury, down-regulated JNK phosphorylation, and inhibited I/R-induced cardiomyocyte apoptosis.

Because ferroptosis can be induced by triggering GPX4 degradation or by treatment with small molecules such as RSL3 that covalently inhibit GPX4 function [41], we further investigated the protection of GPX4 by SAB from the perspective of the GPX4 ubiquitinated proteasomal degradation mechanism. The UPS is an evolutionarily conserved protein degradation and turnover mechanism. This pathway typically consists of three components including the ubiquitin-binding system, deubiquitinases, and the proteasome. Our study found that SAB significantly reversed the ubiquitination of GPX4 induced by H/R stimulation and prolonged the half-life of GPX4 induced by H/R + CHX, thus demonstrating that SAB reduced myocardial cellular susceptibility to MIRI ferroptosis by reducing the ubiquitination-proteasomal degradation of GPX4. Further, pretreatment with the GPX4 inhibitor RSL3 confirmed the protective role of SAB on H/R cardiomyocytes. As expected, the presence of RSL3 essentially abolished the protective role of SAB on H/R cardiomyocytes.

## 4. Materials and Methods

### 4.1. Animals and Grouping

Male Sprague-Dawley rats weighing 200–250 g were obtained from the Laboratory Animal Center of Nanjing Qinglongshan (Nanjing, China). Animals were kept in a temperature and light-controlled room with unlimited water and food. The research was conducted in accordance with the internationally accepted principles for laboratory animal use and care as found in the US guidelines (NIH publication #85–23, revised in 1985). The animal protocol was approved by the Experimental Animal Center of Nanjing University of Chinese Medicine (ACU210706).

The rats were classified into five groups at random (*n* = 12 per group): (1) sham animals treated with saline (control), (2) I/R rats treated with saline (I/R), (3) I/R rats treated with SAB 10 mg/kg (SAB-L), (4) I/R rats treated with SAB 20 mg/kg (SAB-H), and (5) I/R rats treated with diltiazem (Dilt) 20 mg/kg. SAB and diltiazem were administered intraperitoneally at 25 and 1 h before the I/R surgery [42]. SAB (purity ≥ 98%) was purchased from Chengdu Must Bio-technology Co., Ltd. (Chengdu, China). Diltiazem was obtained from Tianjin Tianyu Pharmaceutical Co., Ltd. (Tianjin, China).

### 4.2. Myocardial I/R Surgery

The myocardial I/R process was carried out exactly as mentioned before [43]. Briefly, rats were given 5% isoflurane (pre-anesthesia) to induce anesthesia by inhalation before being maintained on 1.5–2% isoflurane. After tracheotomy, the tracheal cannula was connected to a positive pressure respirator (ALC-V8, Shanghai Alcott Biotech Co., Shanghai, China). The ventilation rate was adjusted to 60–80 breaths/min, with a tidal volume of 2–3 mL/100 g body weight and inspiratory/expiratory ratio of 1:1. Electrocardiogram (ECG) leads were placed on the right foreleg, as well as the right and left hindlegs, to monitor changes in the ST segments throughout the surgery. The left anterior descending coronary artery (LAD) was ligated with a 7–0 silk suture following thoracotomy. After occlusion for 45 min, the coronary suture was released for 2 h to induce reperfusion. The ST-segment elevation on the electrocardiogram was taken as the verification of ischemia. Sham-operated animals underwent the same surgical procedures but did not have their hearts ligated.

### 4.3. The Culture of Cells and the Establishment of H/R Model

The cell bank of the Chinese Academy of Sciences provided the rat cardiomyocyte cell line H9c2. H9c2 cells were grown in a Dulbecco’s modified eagle medium (DMEM) medium with 10% fatal bovine serum (FBS), 100 μg/mL streptomycin, and 100 U/mL penicillin in a humidified incubator (5% CO_2_, 95% air) at 37 °C for normal cell growth.

The H/R cell model was established to imitate the heart ischemia/reperfusion injury in vitro as mentioned before [44,45]. Briefly, the culture medium was changed to a low glucose DMEM medium without FBS, and the cells were cultured in a hypoxic chamber (5% CO_2_, 95% N_2_, 37 °C) for 24 h. Then, cells were reoxygenated for 4 h by utilizing the standard culture method. The oxygen level before and after hypoxia in H9c2 cells were assessed as shown in Figure S3. The H9c2 cells in SAB groups were subjected to SAB pretreatment (10, 20, 40 μg/mL) with or without RSL3((1S,3R)-RSL3) for 24 h before H/R injury.

### 4.4. Measurement of Infarct Size

Evans Blue/TTC dual staining was used to determine the size of the myocardial infarct, as previously reported [46]. To delineate the area at risk, 1 mL of 2% Evans Blue dye was systemically injected into the rat’s circulation system via the femoral vein. Then the rats were sacrificed, and the heart was rapidly removed and kept at −20 °C for 30 min. To visualize the infarct regions, the frozen heart was cut into 2-mm-thick slices and incubated in 1% TTC solution for 20 min at 37 °C. A digital camera was used to photograph the heart slices. The infarct size was determined using the ImageJ software (Version 1.8.0, National Institutes of Health, Bethesda, MD, USA) and presented as a percentage of the area at risk (Inf/AAR%).

### 4.5. Histopathological Examination

The heart was fixed with 4% paraformaldehyde overnight and then embedded in paraffin. Subsequently, 5-μm-thick sections were prepared and stained with H&E (hematoxylin and eosin). A light microscope was used to examine the cardiac histopathological changes.

### 4.6. Western Blot Analysis

The western blot assay was performed as mentioned in previous studies [47]. The cells were seeded into 60 mm dishes at 2 × 10^6^ cells/dish. Protein extracted from cardiac tissues and cells was analyzed by the BCA Protein Assay Kit. SDS-PAGE (Sodium Dodecyl Sulfate—Polyacrylamide Gel Electrophoresis) was used to separate equal amounts of protein, which were then transferred to PVDF membranes. Membranes were incubated with primary antibodies overnight at 4 °C after being blocked with 5% milk. The primary antibodies used included TfR1 (A5865; ABclonal Technology, Woburn, MA, USA), ferritin heavy chain1 (FTH1, 3998; CST, Danvers, MA, USA), GPX4 (ab125066; Abcam, Cambridge, UK), Grsf1(ab205531; Abcam), cleaved Caspase 3 (AF7022; Affinity, West Bridgford, UK), Caspase 3 (AF6311; Affinity), Bax (14796; CST), Bcl2 (ab196495; Abcam), p38 MAPK (14451; CST), phosphop p38 MAPK (p-p38 MAPK;4631; CST), JNK (9252; CST), and phosphop JNK (p-JNK; 4668; CST). The blots were then incubated for 1.5 h with the corresponding secondary antibodies (ab7097; Abcam) and visualized using enhanced chemiluminescence (ECL) and the Bio-Rad imaging system. The protein signals were normalized to glyceraldehyde-3-phosphate dehydrogenase (GAPDH) (ab181602; Abcam). The intensity of protein expression was quantified using ImageJ software.

### 4.7. Co-Immunoprecipitation

The co-immunoprecipitation was performed as mentioned in previous studies [9]. The cells were seeded into 60 mm dishes at 2 × 10^6^ cells/dish. The cells were lysed and incubated with anti-GPX4 overnight at 4 °C. Protein A/protein G-coated agarose beads were then added and incubated for another 4 h at 4 °C. Afterwards, the beads were washed 4 times, boiled for 5 min, and analyzed by immunoblot as above.

### 4.8. Detection of ROS Production

The cells were seeded into 6-well plates at 5 × 10^5^ cells/well. ROS production in heart tissues was detected by dihydroethidium (DHE, Invitrogen, Waltham, MA, USA) staining as previously described [48]. In brief, tissue sections were incubated with 10 μM DHE for 30 min in a light-protected humidified chamber at 37 °C. The fluorescence intensity of DHE was determined using a flow cytometer.

According to the manufacturer’s protocol, a probe 2′,7′-dichlorofluorescein-diacetate (DCF-DA, Sigma, St. Louis, MO, USA) was used to measure the intracellular ROS generation. After treatment with SAB, cells were incubated with 10 μM DCF-DA for 30 min at 37 °C in the dark, and then the fluorescence intensity for DCF-DA was analyzed by a flow cytometer.

### 4.9. Measurements of Malondialdehyde (MDA) and Lactate Dehydrogenase (LDH) Levels

The cells were seeded into 6-well plates at 5 × 10^5^ cells/well. Commercial kits (Nanjing Jiancheng, Nanjing, China) were used to detect the levels of MDA and LDH as directed by the manufacturer.

### 4.10. TUNEL Assay

After the rats were sacrificed, ischemic heart tissue samples were collected, fixed in 10% formalin for 24 h, embedded in paraffin, and cut into 5-μm-thick sections. Assessment for apoptosis was conducted using a commercial apoptosis detection kit (Roche, Basel, Switzerland) according to the protocol described by the manufacturer. Nuclei were stained with DAPI. A fluorescence microscope was used to view the fluorescence staining. The percentage of TUNEL-positive cells in the total cell nuclei was used to calculate the apoptotic index.

### 4.11. Mitochondrial Membrane Potential (MMP, ΔΨm) Detection

The cells were seeded into 24-well plates at 2.5 × 10^4^ cells/well. A JC-1 detection kit (Beyotime, Hong Kong, China) was used according to the manufacturer’s protocol to monitor the change in mitochondrial membrane potential. Red fluorescence indicates JC-1 aggregates in intact mitochondria, whereas green fluorescence indicates JC-1 monomer in apoptotic cells with depolarized mitochondria. A fluorescence microscope was used to examine cells labeled with JC-1.

### 4.12. The MTT Assay

The cells were seeded into 96-well plates at 5 × 10^3^ cells/well and incubated for 24 h. The MTT assay was used to determine cell viability. Then, various concentrations of SAB were added and incubated for 24 h. Afterwards, 10 μL of MTT solution (5 mg/mL) was added and further incubated for 4 h. After removal of the MTT medium, 150 μL of DMSO was added to dissolve the precipitate. The optical density at 570 nm was used to determine cell viability.

### 4.13. Immunofluorescence

The cells were seeded into 48-well plates at 1 × 10^4^ cells/well. The immunofluorescence was performed as previously described [49]. Samples were fixed for 15 min at room temperature in 4% paraformaldehyde and then permeabilized in 0.1% Triton-X for 10 min. Afterwards, 1% bovine serum albumin was used to block the nonspecific sites for 20 min, followed by incubation with anti-GPX4 (DF6701; Affinity) overnight at 4 °C. After washing in PBS, secondary antibodies (ab169346; Affinity) were added to the samples at room temperature for 1 h. Nuclei were stained with DAPI for 5 min in the dark. A fluorescence microscope was used to capture the images.

### 4.14. Cycloheximide Chase Assay

To evaluate whether SAB stabilized the GPX4 protein, cells were treated with 40 μM SAB for 24 h before being exposed to H/R. Then, 50 μg/mL Cycloheximide (CHX) was added and incubated for the indicated time. The half-life of the GPX4 protein was observed by western blot analysis.

### 4.15. Statistical Analysis

All values are expressed as the means ± SD (Standard Deviation). To assess the differences between the groups, data were analyzed using one-way analysis of variance (ANOVA) followed by Dunnett’s or Sidak post hoc tests (GraphPad Software Inc., San Diego, CA, USA). A *p*-value (*p*) < 0.05 was deemed statistically significant.

## 5. Conclusions

**Figure 9:**
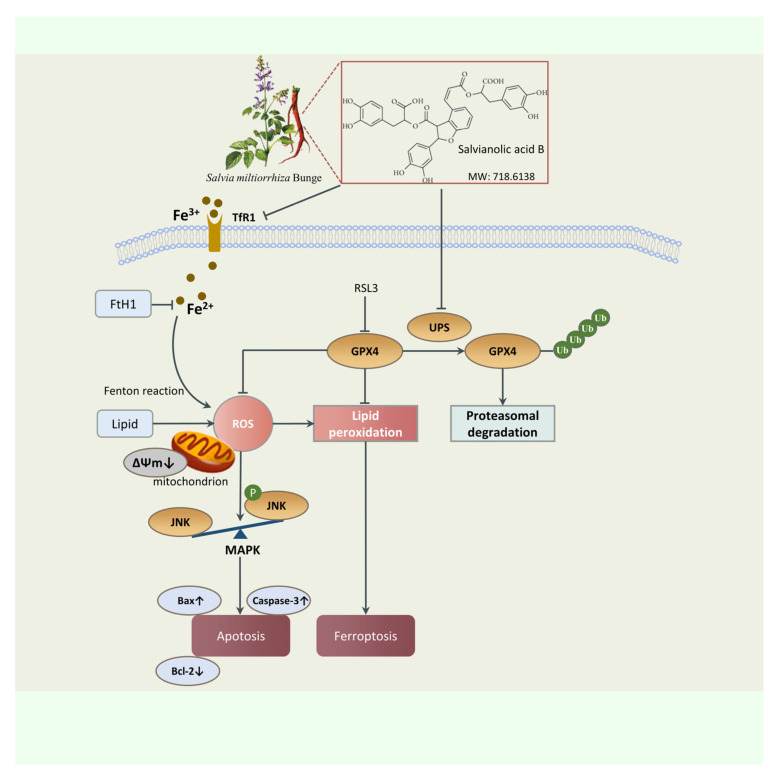
The potential cardioprotection mechanism of SAB via decreasing the ubiquitin-proteasome degradation of GPX4 to inhibit ferroptosis and apoptosis of myocardial ischemia/reperfusion injury (MIRI).

In conclusion, our study revealed that SAB is an effective antioxidant and exerts cardioprotective effects during I/R by anti-oxidative, anti-ferroptotic, and anti-apoptotic effects by reducing ubiquitinated proteasomal degradation of GPX4 and inhibiting ROS-JNK/MAPK signaling (Figure 9). Administration of SAB during I/R may be a potentially effective treatment for clinical acute myocardial infarction.

SAB was found to decrease the ubiquitin-proteasome degradation of GPX4 and formation of reactive oxygen species (ROS), inhibit ferroptosis, activate the JNK/MAPK signal pathway, and cause caspase-associated apoptosis to protect cardiomyocytes from death.

## Data Availability

Data available on reasonable request.

## Supplementary Materials

The following supporting information can be downloaded at: https://www.mdpi.com/article/10.3390/molecules28104117/s1, Figure S1: SAB prevents ferroptosis and apoptosis in H/R-treated isolated adult rat cardiomyocytes. (A) Immunofluorescence staining for GPX4 in isolated adult rat cardiomyocytes. (B) Representative images of JC-1 staining and quantitative analysis of the JC-1 aggregate/monomer fluorescence ratio in isolated adult rat cardiomyocytes. (*n* = 3) Data are presented as means ± SD. ## *p* < 0.01 vs. Control group, ** *p* < 0.01 vs. H/R group; Figure S2: SAB prevents apoptosis in H/R-treated isolated adult rat cardiomyocytes. Western blots and quantitative analysis of p-ERK expression. (*n* = 3) Data are presented as means ± SD. ## *p* < 0.01 vs. Control group, ** *p* < 0.01 vs. H/R group; Figure S3: The oxygen concentration in the cells in hypoxic culture group decreased after 24 h of culture. Intracellular Oxygen Concentration Assay (abcam, ab197245) was to analyse oxygen concentration in the cells. (*n* = 3) Data are presented as means ± SD. ** *p* < 0.01 vs. Control group. Reference [50] is cited in the Supplementary Materials.
