# Supplementary material for: Salvianolic Acid B Inhibits Ferroptosis and Apoptosis during Myocardial Ischemia/Reperfusion Injury via Decreasing the Ubiquitin-Proteasome Degradation of GPX4 and the ROS-JNK/MAPK Pathways
Source: Molecules. 2023 May 16;28(10):4117. doi: 10.3390/molecules28104117 (PMC10224207; doi:10.3390/molecules28104117)
Supplement: Supplementary file 1 [file molecules-28-04117-s001.zip › molecules-2131612-supplementary.pdf]

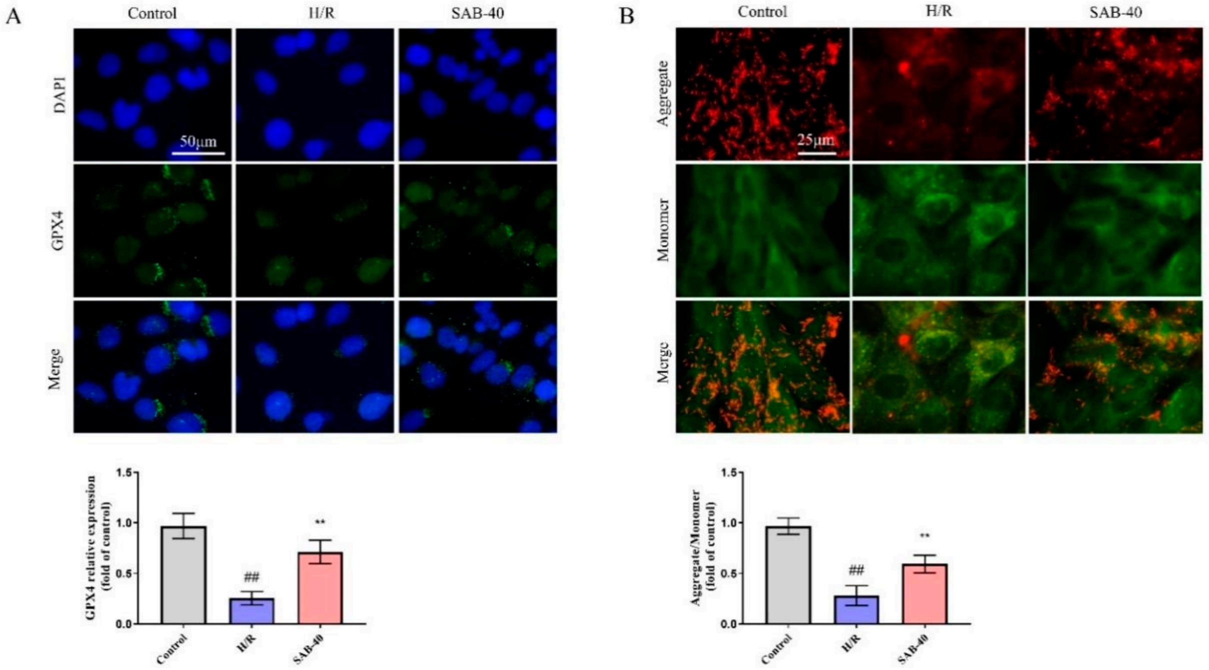

Figure S1 SAB prevents ferroptosis and apoptosis in H/R-treated isolated adult rat cardiomyocytes. (A) Immunofluorescence staining for GPX4 in isolated adult rat cardiomyocytes . (B) Representative images of JC-1 staining and quantitative analysis of the JC-1 aggregate/monomer fluorescence ratio in isolated adult rat cardiomyocytes. (*n*=3) Data are presented as means ± SD. ##*P* < 0.01 vs. Control group, ***P* < 0.01 vs. H/R group

The results show that SAB improved H/R-caused abnormalities in GPX4 fluorescent signal (control: 0.9667±0.1242; H/R: 0.2533±0.06658; SAB-40: 0.7100±0.1153), reflecting its effect on decreasing H/R-triggered ferroptosis (Fig.1A). Meanwhile, the weakened Δψm (control: 0.9667±0.08021; H/R: 0.2800±0.09644; SAB-40: 0.5933±0.08737) was reversed by SAB intervention, suggesting the role of SAB in preventing apoptosis in H/R-exposed isolated adult rat cardiomyocytes.

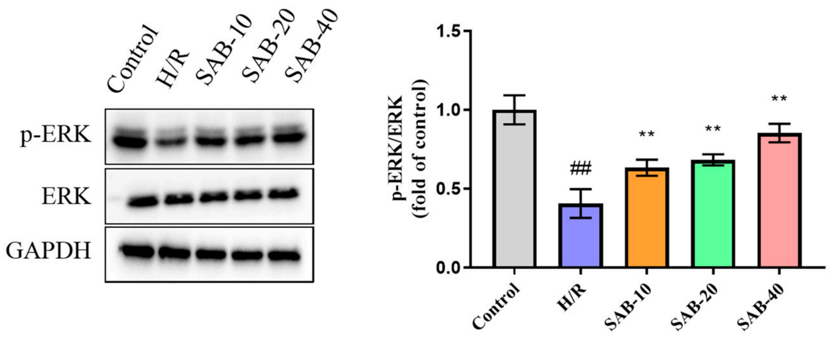

Figure S2 SAB prevents apoptosis in H/R-treated isolated adult rat cardiomyocytes. Western blots and quantitative analysis of p-ERK expression. (*n*=3) Data are presented as means ± SD. ##*P* < 0.01 vs. Control group, ***P* < 0.01 vs. H/R group.

The results show that SAB improved H/R-caused abnormalities in the protein levels of p-ERK/ERK (control: 1.000±0.09165; H/R: 0.4067±0.09074; SAB-10: 0.6333±0.05132; SAB-20: 0.6833±0.03512; SAB-40: 0.8533±0.05859).

Intracellular Oxygen Concentration Assay (abcam， ab197245) was to analyse intracellular oxygen levels before and after hypoxia[1]. Group: normal oxygen culture group (Control) and hypoxic culture group (Model). The oxygen concentration in the cells was measured after 24 hours of culture compared with 0 hours. The result is shown in the following figure:

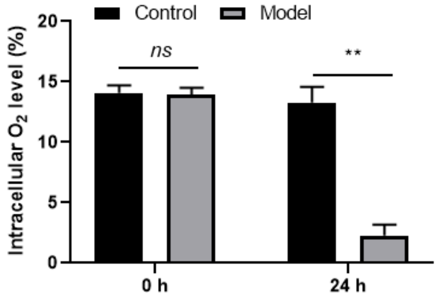

Figure S3: The oxygen concentration in the cells in hypoxic culture group decreased after 24 hours of culture. Intracellular Oxygen Concentration Assay (abcam， ab197245) was to analyse oxygen concentration in the cells. (n=3) Data are presented as means ± SD. **P < 0.01 vs. Control group.

The results showed that after 24 hours of hypoxia culture, the oxygen level in the cells decreased significantly compared with the normal oxygen group (P＜0.01).

References

1. Cubillos-Zapata, C.; Hernández-Jiménez, E.; Avendaño-Ortiz, J.; Toledano, V.; Varela-Serrano, A.; Fernández-Navarro, I.; Casitas, R.; Carpio, C.; Aguirre, L.A.; García-Río, F.; et al. Obstructive Sleep Apnea Monocytes Exhibit High Levels of Vascular Endothelial Growth Factor Secretion, Augmenting Tumor Progression. *Mediators Inflamm.* **2018**, *2018*, 7373921.
